# Thrombosis Despite Sub-Nephrotic Proteinuria

**DOI:** 10.7759/cureus.100192

**Published:** 2025-12-27

**Authors:** Jose Redondo, Alexander J Dobek, Leonardo B Sosa, Rodrigo Santoscoy-Valencia, Efren Manjarrez

**Affiliations:** 1 Internal Medicine, University of Miami Miller School of Medicine, Jackson Memorial Hospital, Miami, USA; 2 Pathology and Laboratory Medicine, University of Miami Miller School of Medicine, Jackson Memorial Hospital, Miami, USA; 3 Hospital Medicine, University of Miami Miller School of Medicine, Jackson Memorial Hospital, Miami, USA

**Keywords:** aa amyloidosis, hypoalbuminemia, peri-procedural anticoagulation, subnephrotic proteinuria, venous thromboembolism (vte)

## Abstract

While nephrotic syndrome is a recognized hypercoagulable state associated with an elevated risk of venous thromboembolism (VTE), sub-nephrotic proteinuria and resultant hypoalbuminemia have also been associated with a similar increased risk.

We report on a 40-year-old patient who initially presented with findings of anasarca and dyspnea and was found to have bilateral lower extremity deep vein thromboses (DVTs) and pulmonary embolism on further testing. In pursuit of a kidney biopsy, the patient’s anticoagulation was briefly held, which resulted in the development of new bilateral upper extremity DVTs.

Our findings outline the need to consider the increased risk for VTE in patients with sub-nephrotic proteinuria and hypoalbuminuria, as the latter finding may be more indicative of clotting risk in this patient population. A multidisciplinary approach is needed to determine the optimal management strategy for these patients, especially when pursuing procedures requiring temporary discontinuation of pharmacological anticoagulation.

## Introduction

Nephrotic syndrome is defined by significant proteinuria (≥3.5 g per 24 hours in adults, or a protein-to-creatinine ratio ≥300-350 mg/mmol), hypoalbuminemia, edema, and hyperlipidemia. It reflects a loss of glomerular permselectivity, which allows large amounts of plasma proteins, particularly albumin and anticoagulant proteins, to be lost in the urine. Primary etiologies include minimal change disease, focal segmental glomerulosclerosis (FSGS), and membranous nephropathy, the latter being most frequently linked to thrombotic complications [1]. Secondary causes include diabetic nephropathy, lupus nephritis (class V), amyloidosis, infectious etiologies (hepatitis B/C, HIV), and drug-induced glomerulopathies [2]. Overall, the unifying pathophysiology is disruption of the glomerular filtration barrier [1,2].

The hypercoagulable state in nephrotic syndrome is a result of a multifactorial disruption in hemostasis. Urinary losses of natural anticoagulants such as antithrombin III, protein C, and protein S diminish endogenous inhibition of coagulation, while hepatic upregulation of procoagulant factors (fibrinogen, factor V, factor VIII, and von Willebrand factor) further shifts the balance toward thrombosis [3-5]. Platelet hyperaggregability, hemoconcentration from intravascular volume depletion, and endothelial dysfunction further augment the prothrombotic physiology [6,7]. Clinically, patients are at increased risk for venous and arterial thromboembolic events, with renal vein thrombosis being particularly characteristic [1]. The risk is greatest in those with severe hypoalbuminemia (<2-2.5 g/dL), membranous nephropathy, or additional thrombotic risk factors [3,8].

Despite extensive literature discussing thrombotic risks in nephrotic syndrome, reports examining the occurrence of VTE in patients with sub-nephrotic-range proteinuria are notably sparse.

Amyloidosis is known to precipitate nephrotic syndrome, and it can be challenging to diagnose due to its often subtle and nonspecific clinical presentation that can overlap with other nephrotic and sub-nephrotic conditions [9]. Consequently, delays in diagnosis are common, potentially exacerbating the risk of serious complications, including thrombosis or bleeding [10]. Furthermore, the existing medical literature lacks comprehensive data clearly delineating the exact threshold of proteinuria and hypoalbuminemia at which thrombotic risk significantly increases. This gap highlights the need for future research to establish clear clinical guidelines for better risk stratification and management of these patients.

Epidemiological studies indicate that approximately 5-10% of patients with amyloidosis experience thrombotic events, with particular prominence in those with cardiac involvement, further complicating clinical management [11,12]. Specifically, nephrotic-range proteinuria with hypoalbuminemia (albumin <3 g/dL) significantly increases thrombotic risk, as demonstrated by hazard ratios of 4.3 or higher compared with patients with higher serum albumin levels [11]. Moreover, cardiac amyloidosis, characterized by atrial fibrillation and restrictive cardiomyopathy, also notably increases thromboembolic risk [13].

Clinicians face significant management challenges due to the delicate balance required between anticoagulation for thrombotic risks and heightened bleeding risks inherent to systemic amyloidosis, including factor X deficiency, vascular fragility, renal impairment, and gastrointestinal amyloid involvement [13]. Enhanced understanding and further reporting of thrombotic complications in subnephrotic proteinuria cases will facilitate better risk stratification, timely diagnosis, and informed therapeutic decision-making.

## Case presentation

A 40-year-old female with a medical history significant for hypertension, opioid and sedative use disorders, mood disorder, recurrent cellulitis related to intravenous injection sites, and hepatitis C (currently with undetectable viral load) presented to the emergency department with a three-week history of progressively worsening generalized edema (anasarca). Initially, the swelling was limited to the lower extremities but gradually progressed, causing shortness of breath during routine daily activities. The patient disclosed ongoing intravenous fentanyl use, last administered on the morning prior to admission, typically injecting into her legs and hips.

Upon examination, she exhibited severe generalized edema, particularly pronounced in the lower extremities, and demonstrated exertional dyspnea. There was significant leukocytosis initially suspected to be related to either a chronic right hip ulcer or colitis; however, the ulcer appeared well-healed with no active signs of infection.

**Figure 1:**
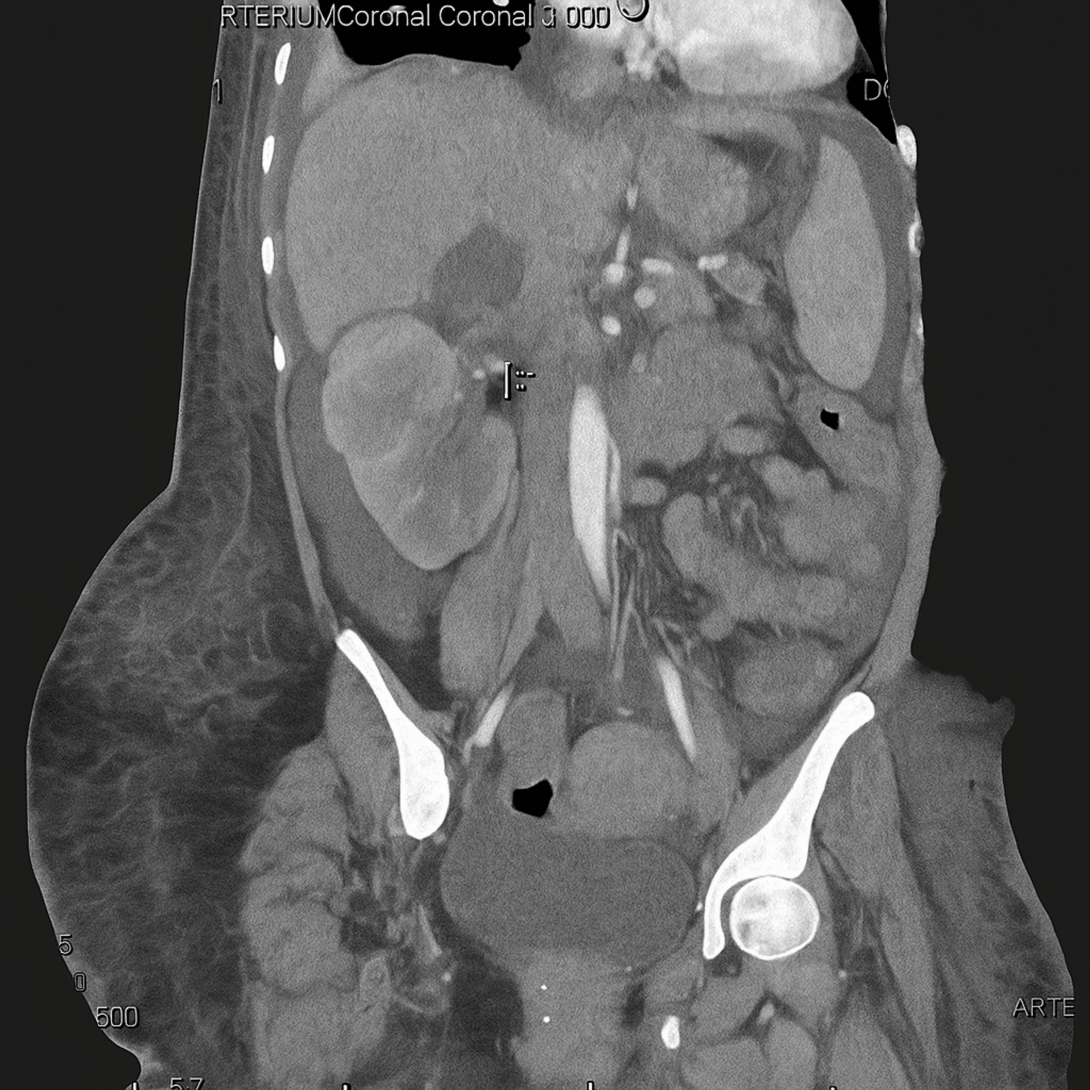
Renal vein thrombosis—a frequent site of thrombi formation in patients with nephrotic syndrome.

**Figure 2:**
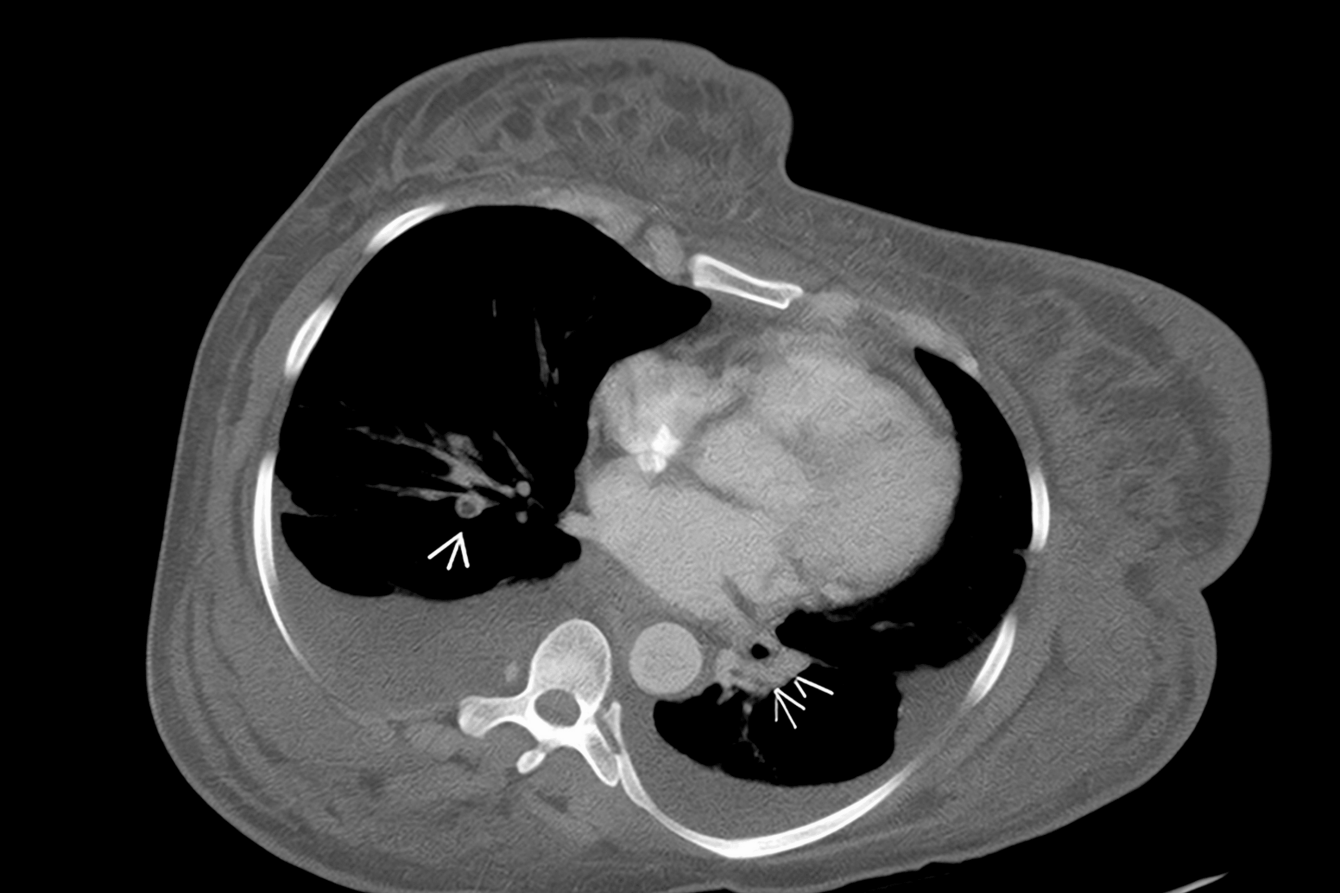
Bilateral acute segmental pulmonary emboli at the lung bases.

Given the clinical suspicion of venous thromboembolism (VTE), a CT chest was performed, revealing bilateral renal vein thrombosis and pulmonary emboli (Figures 1, 2). Consequently, therapeutic anticoagulation was initiated. Additional positive pertinent workup is illustrated in Table 1. Antiphospholipid antibody testing was negative, and serum protein electrophoresis showed no evidence of multiple myeloma.

**Table 1:** Summary of laboratory findings and reference ranges.

Test	Results	Normal Range	Interpretation
24-hour Urine Protein	2.8 g/day	< 150 mg/day (normal); nephrotic range > 3.5 g/day	Elevated (sub-nephrotic range)
Serum Albumin	1.1 – 1.6 g/dL	3.5 – 5.0 g/dL	Markedly decreased (severe hypoalbuminemia)
Triglycerides	250 mg/dL	< 150 mg/dL	Elevated (hypertriglyceridemia)
C-reactive Protein (CRP)	2.4 mg/dL	< 3 mg/L (≈ < 0.3 mg/dL)	Mildly elevated
Antinuclear Antibody (ANA)	1:80 (fine granular pattern)	Negative (< 1:40)	Low-positive

**Figure 3:**
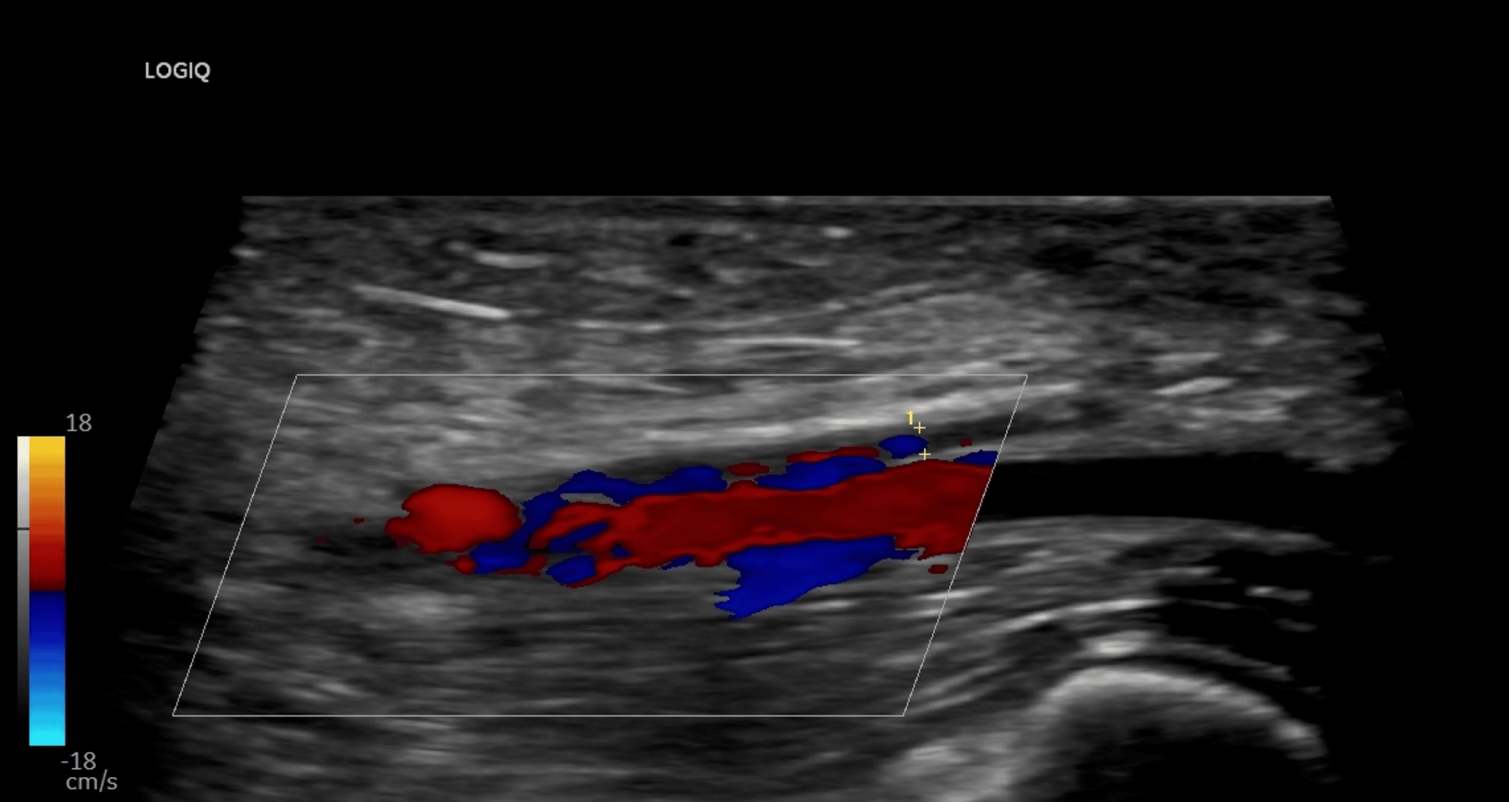
DVT of the right brachial vein—formed in less than 24 hours after stopping anticoagulation. This underscores the hypercoagulability state in this patient population with serum hypoalbuminemia and nephrotic-range proteinuria. DVT: deep vein thromboses

**Figure 4:**
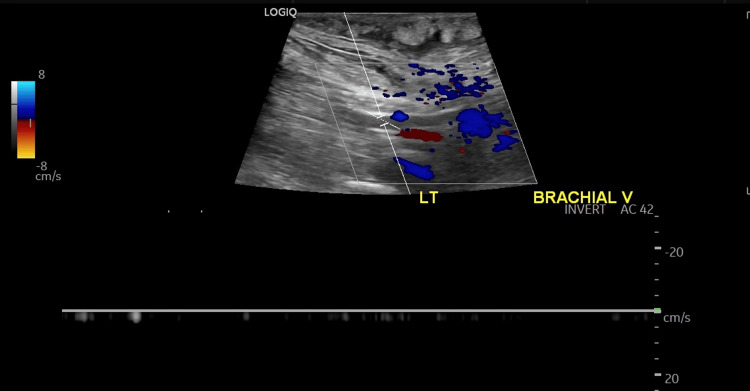
DVT of the left brachial vein formed in less than 24 hours after stopping anticoagulation. This underscores the hypercoagulability state in this patient population with serum hypoalbuminemia and nephrotic-range proteinuria. DVT: deep vein thromboses

**Figure 5:**
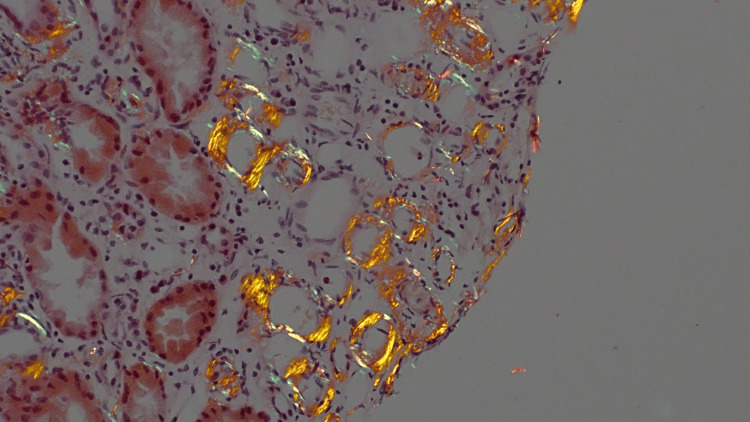
Deposition of acellular, amorphous, pale eosinophilic material in the vasa recta, which shows characteristic apple-green birefringence under polarized light on Congo red stain by light microscopy. These findings are diagnostic of amyloidosis and reveal the likely etiology for the patient's hypercoagulable state.

Due to the unclear etiology of proteinuria, a diagnostic renal biopsy was obtained. The heparin drip was paused peri-procedurally (overnight and for 8 hours post-procedure). During this time, the patient developed acute bilateral upper extremity swelling. Ultrasound confirmed extensive bilateral upper extremity DVTs (Figure 3) (Figure 4). Anticoagulation was resumed and continued throughout hospitalization. Upon discharge against medical advice, the patient was prescribed apixaban. Findings of renal biopsy were consistent with AA amyloidosis, showing characteristic apple-green birefringence under polarized light on Congo red staining (Figure 5).

## Discussion

This case highlights a rare but clinically significant scenario: the development of extensive VTE in a patient with sub-nephrotic-range proteinuria but profound hypoalbuminemia, ultimately diagnosed with AA amyloidosis. While nephrotic-range proteinuria is a well-established risk factor for thrombotic complications, our case adds to growing evidence that severe hypoalbuminemia, regardless of proteinuria threshold, may serve as a more sensitive marker of hypercoagulability and VTE risk in glomerular diseases.

In a large cohort study of patients with AL amyloidosis, hypoalbuminemia was shown to have a greater predictive value for the development of VTEs when compared to proteinuria alone [11]. This is reinforced in our case, where the patient had a 24-hour urine protein of 2.8 g/day-below the nephrotic threshold, along with significant serum hypoalbuminemia, ranging from 1.1 to 1.6 g/dL. In this case, the patient subsequently developed extensive upper extremity DVTs during a short interruption in anticoagulation (less than 24 hours).

This scenario draws attention to the potential shortcomings of relying solely on proteinuria levels to stratify thrombotic risk in patients with identified severe proteinuria, often seen in nephrotic syndrome. The pathophysiologic underpinnings likely involve urinary loss of antithrombotic proteins such as antithrombin III and plasminogen, which correlates more closely with hypoalbuminemia than with proteinuria [14]. Hypoalbuminemia may also promote hypercoagulability via increased hepatic synthesis of prothrombotic factors and enhanced platelet aggregation [15].

In most patients with nephrotic-range proteinuria without a clear etiology, a renal biopsy should be considered to evaluate for glomerulonephropathies. This diagnostic step was done in the course of this patient's hospital stay, weighing the potential thrombotic risk, and the diagnosis of AA amyloidosis was made, as seen in Figure 4. In this case, as in most cases, it is impossible for a clinician to be certain of an underlying condition such as amyloidosis, which may pose an additional prothrombotic risk, before a renal biopsy is completed. So, unless there is additional organ involvement, which may be suspicious for amyloidosis, a clinician would have no reason to suspect it, and in turn may be less concerned for the development of thrombi.

Epidemiological studies indicate that approximately 5-10% of patients with amyloidosis experience thrombotic events, with particular prominence in those with cardiac involvement, further complicating clinical management [11,12]. Specifically, nephrotic-range proteinuria with hypoalbuminemia (albumin <3 g/dL) significantly increases thrombotic risk, as demonstrated by hazard ratios nearing 4.3 compared to patients with higher albumin levels [4]. Moreover, cardiac amyloidosis, characterized by atrial fibrillation and restrictive cardiomyopathy, also notably increases thromboembolic risk [12].

Importantly, our case also illustrates the precipitous formation of new thrombi during a brief interruption of therapeutic anticoagulation, a clinical situation frequently encountered in the hospital setting. Anticoagulation was held for less than 24 hours for a diagnostic renal biopsy, yet the patient developed extensive bilateral upper extremity DVTs. This observation emphasizes that even transient pauses in anticoagulation can be enough to tip the balance toward thrombosis in high-risk patients, especially those with systemic amyloidosis, profound hypoalbuminemia, or existing thrombotic events.

More confounding is that patients with amyloidosis may also have bleeding diatheses, including acquired factor X deficiency, amyloid-related vascular fragility, and gastrointestinal amyloid deposition [16]. This further complicates the risk-benefit assessment of anticoagulation. Thus, clinicians often find themselves in a catch-22, as in our case: needing to interrupt anticoagulation for invasive procedures like renal biopsy (essential for diagnosis and treatment planning) while simultaneously risking life-threatening thrombosis during that interruption. This case exemplifies that dilemma and underscores the need for more nuanced clinical pathways.

Risk stratification tools for VTE in glomerular disease and amyloidosis remain unexplored. Traditional coagulation markers (e.g., D-dimer, PTT, fibrinogen) have not consistently predicted thrombotic risk in amyloidosis cohorts. Serum albumin may serve as a more accessible and practical marker for identifying high-risk patients who could benefit from more aggressive prophylactic anticoagulation or alternative procedural planning.

This case also raises questions about optimal anticoagulation management around procedures in patients with systemic amyloidosis. There is an urgent need for evidence-based peri-procedural anticoagulation protocols tailored to this population. In the interim, multidisciplinary approaches involving nephrology, hematology, and procedural teams are crucial to weigh the risks and timing of holding anticoagulation for diagnostic biopsies.

## Conclusions

This case highlights an uncommon yet clinically consequential presentation of extensive venous thromboembolism occurring in the setting of sub-nephrotic-range proteinuria with profound hypoalbuminemia, ultimately attributable to AA amyloidosis. While nephrotic-range proteinuria has traditionally guided thrombotic risk stratification in glomerular disease, our case reinforces emerging evidence that severe hypoalbuminemia may be a more sensitive and clinically relevant predictor of hypercoagulability, even when proteinuria falls below conventional nephrotic thresholds. The rapid development of extensive upper extremity deep vein thromboses during a brief (<24-hour) interruption of anticoagulation underscores the fragile hemostatic balance in patients with systemic amyloidosis and marked hypoalbuminemia. This observation is particularly salient given the frequent need to temporarily hold anticoagulation for diagnostic procedures such as renal biopsy, often essential for definitive diagnosis, yet potentially catastrophic in high-risk individuals. 

Our case emphasizes several important clinical implications. First, clinicians should maintain a high index of suspicion for thrombotic risk in patients with profound hypoalbuminemia, even in the absence of nephrotic-range proteinuria. Second, serum albumin may represent a practical and underutilized marker for identifying patients at heightened thrombotic risk who may warrant intensified monitoring, early anticoagulation, or alternative procedural strategies. Finally, this case highlights a critical gap in current practice: the lack of evidence-based, peri-procedural anticoagulation guidelines tailored to patients with amyloidosis and glomerular disease.
